# Increasing incubation periods during a prolonged monophasic *Salmonella* Typhimurium outbreak with environmental contamination of a commercial kitchen at Oslo Airport, Norway, 2017

**DOI:** 10.2807/1560-7917.ES.2019.24.34.1900207

**Published:** 2019-08-22

**Authors:** Lotta Siira, Emily MacDonald, Gry Marianne Holmbakken, Tom Sundar, Lars Meyer-Myklestad, Heidi Lange, Lin T Brandal, Umaer Naseer, Gro S Johannessen, Bjarne Bergsjø, Laura Espenhain, Line Vold, Karin Nygård

**Affiliations:** 1 Norwegian Institute of Public Health, Oslo, Norway; 2 European Program for Public Health Microbiology Training (EUPHEM), European Centre for Disease Prevention and Control, (ECDC), Stockholm, Sweden; 3 Norwegian Food Safety Authority, Oslo, Norway; 4 Municipality of Nannestad, Akershus, Norway; 5 Municipality of Ullensaker, Akershus, Norway; 6 Norwegian Veterinary Institute, Oslo, Norway; 7 European Programme for Intervention Epidemiology Training (EPIET), European Centre for Disease Prevention and Control, (ECDC), Stockholm, Sweden

**Keywords:** food-borne diseases, disease outbreaks, *Salmonella* Typhimurium, monophasic *Salmonella* Typhimurium, Norway, whole genome sequencing

## Abstract

In September 2017, a cluster of monophasic *Salmonella* Typhimurium isolates was identified at the National Reference Laboratory for Enteropathogenic Bacteria in Norway. We investigated the cluster to identify the source and implement control measures. We defined a case as a person with laboratory-confirmed salmonellosis with the outbreak strain multiple locus variable-number tandem repeat analysis type. We conducted descriptive epidemiological and environmental investigations and performed whole genome sequencing (WGS) with core and accessory genome multilocus sequence typing of all isolates from cases or the environment connected with this outbreak. We identified 21 cases, residing in 10 geographically dispersed counties, all of whom had consumed food or drinks from a café at Oslo Airport. Case distribution by date of symptom onset suggested that a point source was introduced in mid-August followed by continued environmental contamination. The incubation periods ranged 0–16 days and increased as the outbreak progressed, likely due to increasingly low-dose exposure as control measures were implemented. WGS confirmed an identical cluster type-944 in all cases and six environmental specimens from the café. Control measures, including temporary closure and kitchen refurbishment, failed to eliminate the environmental source. We recommend strengthened hygiene measures for established environmental contamination during an outbreak.

## Background

Non-typhoidal *Salmonella* infection is the second most commonly reported gastrointestinal infection in the European Union/European Economic Area (EU/EEA) [1]. In Norway, *Salmonella* infections have been notifiable to the Norwegian Surveillance System for Communicable Diseases (MSIS; http://www.msis.no/) since 1975. Since 2005, between 866 and 1,942 cases of salmonellosis have been reported annually. Typically, 60–80% of notified cases are acquired abroad, as Norway has few established domestic *Salmonella* reservoirs. *S.* Typhimurium is one of the most commonly encountered *Salmonella* serovars in Norway accounting for 33% of domestically acquired salmonellosis cases in 2000-15 [2]. In the past 20 years, monophasic *S.* Typhimurium with antigenic formula 4,[5]12:i.- has been identified in several countries, both in production animals and human cases [2-4]; monophasic *S.* Typhimurium is especially associated with pig production [5,6]. In Europe, the first described monophasic *S.* Typhimurium outbreak was caused by a ‘Spanish clone’, which emerged in 1997 [7]. In Norway, monophasic *S.* Typhimurium was first identified in 2007 and in 2016, it accounted for 12% of all salmonellosis cases and 17% of all domestic salmonellosis cases reported to the Norwegian Institute for Public Health (NIPH) [8].

The incubation period of salmonellosis is typically between 6 and 72 hours, but incubation periods of up to 16 days have been documented following low-dose exposure [9-11]. The infectious dose for salmonellosis varies by serovar but is usually > 10^5^ bacteria [12]. Symptoms of non-typhoidal salmonellosis are diarrhoea, nausea, headache and abdominal cramps. Fever may also be present [13].

### Outbreak detection

On Friday 15 September 2017, the National Reference Laboratory (NRL) for Enteropathogenic Bacteria for human matrices at NIPH reported a cluster of six monophasic *S.* Typhimurium isolates sharing a rare multiple locus variable-number tandem repeat analysis (MLVA) type (3-13-12-NA-210). The cases resided in five geographically dispersed municipalities in Norway and no travel abroad was reported in the week before symptom onset. NIPH initiated an outbreak investigation in collaboration with the Norwegian Food Safety Authority (NFSA) and the municipal medical officers in the affected municipalities to identify the source of the outbreak in order to implement control measures and prevent further spread.

## Methods

### Case definition

For this outbreak, a case was defined as a person residing in Norway with a laboratory-confirmed infection with monophasic *S.* Typhimurium MLVA type 3-13-12-NA-210 sampled after 15 August 2017.

### Case finding

In Norway, all *Salmonella* isolates are submitted by the medical microbiology laboratories to NRL at NIPH for confirmation and characterisation.

On 15 October 2017, the NIPH requested, through the Epidemic Intelligence Information System (EPIS) coordinated by the European Centre for Disease Prevention and Control (ECDC), information on whether other countries had identified cases of monophasic *S.* Typhimurium with the outbreak MLVA type.

### Epidemiological investigation

Four initial cases were interviewed using a standardised 19-page *Salmonella*-specific trawling questionnaire [14]. The questionnaire included detailed questions about food consumption and purchases, animal contact and environmental exposures in the week before the onset of symptoms, as well as clinical and demographic information. Following analysis of information from these interviews, the questionnaire was shortened to focus on categories of most interest, which included domestic travel before symptom onset and food items consumed at the cafés at Oslo Airport. The interviews were carried out by municipal medical officers in the municipality of the cases or by NFSA and NIPH staff.

A descriptive analysis of the cases and results of the interviews was conducted using Excel 2013 and STATA v15 (StataCorp, College Station, Texas, United States). The mean incubation periods of cases in the periods of August 2017 and September 2017 onwards were compared by t-test.

## Microbiological investigation

### Human specimens

In Norway, all faecal specimens from patients with gastroenteritis are routinely analysed for at least *Salmonella, Campylobacter, Shigella* and *Yersinia* at the medical microbiology laboratories. *Salmonella* isolates are submitted to the NRL at NIPH, where they are serotyped by agglutination tests with antisera (SIFIN, Berlin, Germany and Statens Serum Institut (SSI), Hillerød, Denmark) according to the White-Kauffmann scheme [15] and MLVA typed as described previously [16]. The isolates were tested for susceptibility to ampicillin, azithromycin, cefotaxime, gentamicin, meropenem, pefloxacin, tetracycline, and trimethoprim-sulphamethoxazole according to the European Committee on Antimicrobial Susceptibility Testing (EUCAST) 7.1 guidelines (http://www.eucast.org). Where EUCAST breakpoints were not available, epidemiological cut-off values were used based on national zone distributions (tetracycline: R < 17 mm) [17].

### Environmental and food specimens

Environmental swabs and food specimens were collected by NFSA during site inspection on 22 September and environmental swabs on 21 November 2017. These were analysed at the Norwegian Veterinary Institute, NRL-Salmonella for non-human matrices, for the presence of *Salmonella* and *Escherichia coli*. Monophasic *S.* Typhimurium variants were identified according to the European Food Safety Authority (EFSA) recommendations [18] and sent to the NRL at NIPH for further typing as described above.

In the period 13–23 October, environmental samples collected by the company operating the café were analysed at a private laboratory and sent to the Norwegian Veterinary Institute for verification and then submitted to the NRL at NIPH for typing as described above.

### Comparison of isolates

All human and environmental monophasic *S.* Typhimurium isolates connected with the outbreak were analysed at NIPH by whole genome sequencing (WGS). The raw reads were submitted to the European Nt Archive (ENA) under the accession numbers ERS3466858-ERS3466884. Classical multilocus sequence typing (MLST, 7 loci) core genome (cgMLST, 3,002 loci) and accessory genome MLST (aMLST, 1,324 loci) containing 4,333 loci was performed on WGS data. The cgMLST was performed as previously described [19]; for the in-house aMLST *S.* Typhimurium TW-Stm6 (CP019649.1) was used as a reference genome.

The online tool ResFinder version 3.0, available at the Center for Genomic Epidemiology (http://www.genomicepidemiology.org/) was used for sequence-based identification of acquired resistance genes using assembled genomes obtained through SPAdes Genome Assembler version 3.0 (Algorithmic Biology Laboratory, St. Petersburg University, St. Petersburg, Russia) [20]. Default threshold values were used.

### Environmental investigation

The NFSA inspected the café kitchen on 20 and 22 September and 21 November 2017, swabs from kitchen surfaces were collected during the two latter inspections. NFSA also reviewed documentation for suspected food products delivered to the implicated café.

During the period 13 October–15 November, the company operating the café collected environmental specimens on nine occasions.

### Ethical statement

Ethical approval was not required as outbreak investigations are covered under national legislation. Cases were asked for consent to participate at the start of the interviews.

## Results

### Epidemiological investigation

#### Description of the cases

As at 1 February 2018, 21 confirmed cases were reported to the NIPH. Thirteen (13/21) were women and the median age was 27 years (range 17–60) (Table). The cases resided in 10 geographically dispersed counties in Norway. International requests returned no reports of cases in other European countries.

**Table:** Description of identified cases in the outbreak of monophasic *Salmonella* Typhimurium MLVA type 3-13-12-NA-210, associated with a café at Oslo Airport, Norway, 2017 (n = 21)

Age group (years)	Number of cases		
	Male	Female	Total
< 20	1	2	3
20–29	5	4	9
30–39	2	2	4
≥ 40	0	5	5
Total	8	13	21

**Figure 1:**
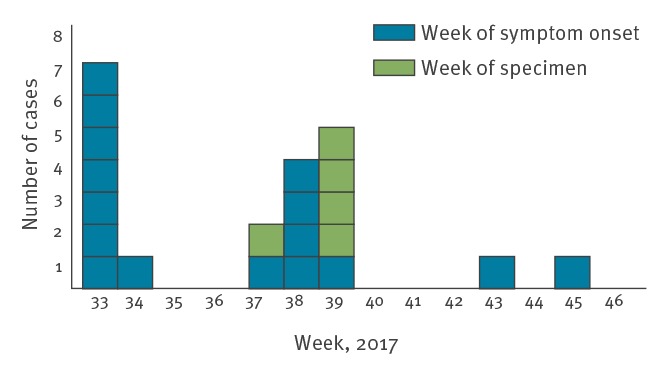
Cases infected with monophasic *Salmonella* Typhimurium MLVA type 3-13-12-NA-210 by week of symptom onset^a^ or week of specimen collection^b^, café at Oslo Airport, Norway, August–November 2017 (n = 21)

^a^ Week of symptom onset (n = 16).

^b^ Week of specimen collection (n = 5).

**Figure 2:**
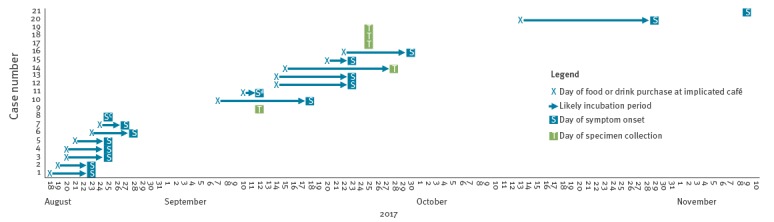
Outbreak timeline with incubation periods for cases of monophasic *Salmonella* Typhimurium MLVA type 3-13-12-NA-210 infection by date of symptom onset^a^ or specimen collection date^b^, café at Oslo Airport, Norway, 2017 (n = 21)

^a^ Week of symptom onset (n = 16).

^b^ Week of specimen collection (n = 5).

^c^ Same day of exposure and symptom onset.

^d^ Uncertain day of onset.

Date of symptom onset was available for 16 cases and ranged from 23 August to 18 November 2017, with the majority occurring in the first week of the outbreak (Figure 1). For the 15 cases with available date of symptom onset and date of exposure, the incubation period ranged between 0 and 16 days and increased as the outbreak progressed (p = 0.029) (Figure 2). The median incubation period in August was 4.5 days (range 0–5) and 9 days (range 2–16) from September onwards.

Week of specimen collection is shown where week of symptom onset was not available.

Four cases were interviewed with the *Salmonella*-specific trawling questionnaire and the remaining cases with a more focused questionnaire. All cases interviewed with the trawling questionnaire had visited Oslo Airport and had consumed different food or drink items at the same café. In total, all 21 cases reported having consumed food or drink items at the café between 18 August and 13 October 2017. The cases reported consuming at least three different types of sandwiches and seven different fruit or vegetable juices; no single common food or drink item was reported by all interviewed cases. No other common exposures at Oslo Airport were identified. The cases included members of staff at the implicated café.

## Microbiological investigation

Twenty-one human monophasic *S.* Typhimurium isolates with the MLVA type 3-13-12-NA-210 were identified by the NRL at NIPH.

Six environmental specimens collected from a kitchen drain, water tap and a wall mounted steel shelf were positive for the outbreak strain. All 10 collected food specimens tested negative for *Salmonella.*

**Figure 3:**
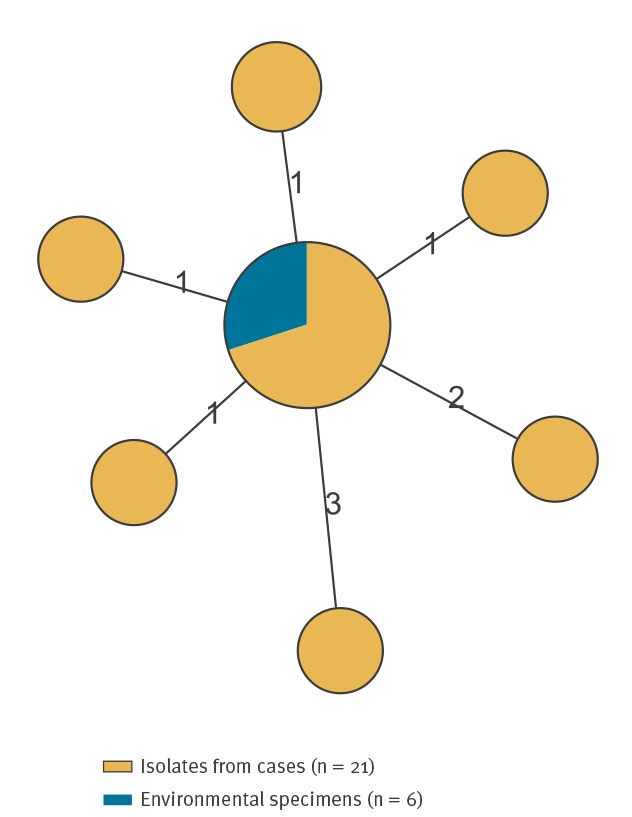
Minimum spanning tree of monophasic *Salmonella* Typhimurium MLVA type 3-13-12-NA-210 isolates from human and environmental specimens, café at Oslo Airport, Norway 2017

All isolates of human and environmental origin were sequence type (ST) 34 and cluster type (CT) 944. The isolates clustered together and cgMLST and aMLST showed that there were three or fewer allelic differences between the isolates (Figure 3).

Isolates were characterised by core and accessory gene multilocus sequence typing including 4,333 loci. Numbers on branches denote allelic differences between isolates.

The outbreak strain was resistant to ampicillin and tetracycline and carried the *bla*_TEM-1B_ and *tet*B genes that confer resistance to β-lactams and tetracycline, respectively.

## Environmental and trace-back investigation

The company operating the café provided documentation on wholesalers where they had sourced food items; some of them were located outside Norway. NFSA received documentation on foods obtained from wholesalers in the period 9–25 August, as well as on 21 September and 26 September 2017. The review of documentation connected to food batches delivered to the café was inconclusive.

The inspections identified several weaknesses in the hygiene routines at the café, including separation between clean and unclean kitchen areas, use of appropriate detergents for different cleaning purposes and which basins that should be used to wash hands, food items and dishes, respectively. Furthermore, the washing routines of workwear was not optimal and there was lacking assessment and management of potential risks connected with the work processes and ingredients.

## Outbreak control measures

**Figure 4:**
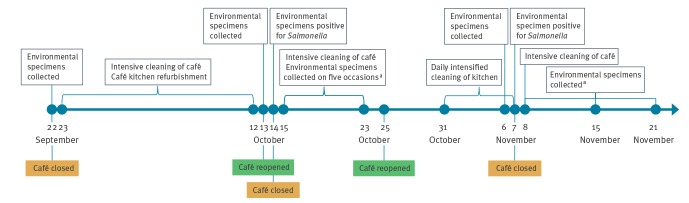
Flowchart of the control measures taken during the outbreak of monophasic *Salmonella* Typhimurium, café at Oslo airport, Norway, 2017

^a^ Environmental specimens that tested negative for the outbreak strain *Salmonella* Typhimurium MLVA type 3-13-12-NA-210.

The timeline for outbreak control measures can be seen in Figure 4.

On 22 September, the café closed temporarily to wash down the facilities and replace some of the kitchen equipment, while awaiting results from environmental specimens collected the same day. The café reopened on 13 October and simultaneously engaged a private company to collect environmental specimens. The next day the café was informed that preliminary results from some specimens showed the presence of *Salmonella* and it closed again for an additional wash down. New specimens were collected on five occasions 15–23 October and the facilities were cleaned intensively several times during this period. On 25 October, the café reopened. According to the company operating the café, the staff that reported for work had tested negative for *Salmonella*, five rounds of negative environmental specimens had been analysed and all inventory and kitchen equipment had been replaced. The company contracted cleaning staff to perform daily cleaning of all contact points and surfaces between 31 October and 7 November and had environmental specimens collected 6 November, while the café was open for business. On 7 November, one specimen with suspected *Salmonella* was reported among those collected the previous day and the café was closed again. The suspected specimen had been collected from a shelf in the kitchen that had previously tested negative and had been installed after the inspection by NFSA on 22 September. Following the suspected finding, decontamination was undertaken and new specimens were collected on 8 and 15 November. These specimens tested negative. Environmental specimens collected by NFSA on 21 November were also negative.

## Discussion

The dispersion of monophasic *S.* Typhimurium MLVA type 3-13-12-NA-210 cases around Norway initially suggested an outbreak linked to a nationally distributed product. Interviews of cases and descriptive epidemiology revealed that all cases had travelled by air or visited Oslo Airport in the days before illness onset, linking the outbreak to a local source. The results from the microbiological investigation of human and environmental specimens showed that the monophasic *S.* Typhimurium isolates were of the same MLVA type and clustered together closely in WGS analysis, which pointed to a café at Oslo Airport as the common source of the outbreak. This outbreak was likely associated with consumption of food or drink items from this café, which was located outside the security restricted area in the terminal and therefore accessible to anyone visiting the airport. Trawling interviews and descriptive epidemiology, in conjunction with environmental and microbiological investigation, were sufficient to identify the location of the source. This outbreak illustrates how WGS can be used to link patient and environmental specimens in an outbreak investigation context. Any suspected outbreak strains were analysed by WGS based cgMLST and aMLST, as they were received at the NRL at NIPH, which allowed for timely and ongoing confirmation of cases and isolates to be linked to the outbreak with high resolution.

The dates of onset of the first cases between 22 and 28 August suggested that the outbreak was caused by a point source, likely introduced at the café in mid-August. During the next several weeks the café was open for business, which resulted in ongoing exposure for customers and staff. The duration of this outbreak from August until November and the increasing incubation period over this time suggested that the source was environmental i.e. customers were continuously exposed but the dose of *Salmonella* decreased over time. Environmental contamination has previously been implicated in a protracted *S.* Typhimurium outbreak in the United Kingdom; cases were identified over a period of more than 13 months and aerosolised drain contamination from a restaurant kitchen drainage system was suspected as the source of transmission [21]. Previous studies have also indicated that low-dose exposure is associated with a prolonged incubation period [9-11], which would fit with the hypothesis of a lower dose obtained through contamination of food items through an environmental reservoir. As salmonellosis cases are normally interviewed about their exposures in the week before symptom onset for hypothesis generation, investigations of low-dose exposure outbreaks may miss exposures outside the standard incubation period.

### Control measures

The company operating the Oslo Airport café undertook extensive control measures, including several periods of voluntary closure, refurbishment of the kitchen and deep cleaning, however, eliminating the source of the environmental contamination proved difficult. This was likely due to the environmental contamination with specimens taken from several sites e.g. a kitchen drain, a tap and a steel shelf, all testing positive for the outbreak strain. The environmental inspection revealed several weaknesses in kitchen hygiene and food handling routines, possibly facilitating the cross-contamination of food items and protraction of the outbreak. This outbreak highlights the importance of ensuring food handlers are provided with adequate training to adhere to strict hygiene measures and routines throughout food handling in order to avoid environmental contamination. In addition to these food handling and hygiene practices, intensified hygiene audits and regular environmental specimen collection could be considered, especially in a context where large quantities of raw fruits and vegetables are processed in a commercial kitchen. Follow up with cleaning companies is also important to ensure wash downs are conducted as planned.

### Source of infection

We are unable to conclude how the source of infection was introduced at the café. We foresee two possible hypotheses; the source of infection was either introduced via a contaminated food item, or via infected staff.

For the hypothesis that the source of infection was introduced by a contaminated food item, there are several points to consider. First, while the entire chain of cafés sources their ingredients from the same wholesalers, no other cafés operated by this company in Norway (or in other countries) were linked to similar infections. However, it is known from the literature that partial food batches can be contaminated and it is not always known at which point the contamination has occurred [22,23]. Second, the cases reported consuming different food and drink items from the café, making it less likely that one contaminated food item was the direct source of infection for all the cases. It is possible, however, that one product introduced the pathogen into the kitchen environment. Third, the outbreak was prolonged, even though the foods handled at the café were fresh produce with a short shelf life. Finally, the outbreak strain was not detected in food specimens collected at the café after the start of the outbreak; it is worth noting, however, that food items from the batches used in August were not available at the time of specimen collection. Detection of the outbreak strain in environmental specimens indicates that the contamination of the café was extensive. The hypothesis of introduction through a contaminated food item is supported by how the fresh produce was processed at the café, as the environmental inspection revealed the risk of cross-contamination.

Some evidence supports the hypothesis of infected staff as the source of introduction. First, the outbreak strain was present in specimens obtained from some staff members, which indicates that the strain circulated among the staff. However, staff members reported that they had consumed food items from the café, so they may have been exposed at work. Second, after being notified of the outbreak, the company operating the café required all staff members to provide a negative stool specimen before they could report to work; some asymptomatic staff members were identified this way. Finally, information obtained from the company operating the café shows staff members may have been ill in the run-up to the start of the outbreak. However, it is unknown whether any previous illness was caused by the outbreak strain.

### Conclusions and recommendations

This common source monophasic *S.* Typhimurium MLVA type 3-13-12-NA-210 outbreak at Oslo Airport has implications beyond the local setting, as cases resided in several dispersed counties across the country. Although our EPIS request returned no reports of international cases, an outbreak at an international airport could easily have geographically wider implications. Furthermore, the café, where large quantities of fresh ingredients are processed, is part of an international chain with the company operating cafés in at least seven European countries, in addition to countries outside Europe.

The epidemiological and microbiological results from cases and environmental specimens obtained at the café support the hypothesis of a common source monophasic *S.* Typhimurium MLVA type 3-13-12-NA-210 outbreak. No further cases or isolates of the outbreak strain were identified at the NRL at NIPH after 18 December 2017, which indicates that the control measures that were implemented, including the voluntary closure of the café, successfully ended the outbreak. We recommend molecular surveillance for outbreak detection and investigation, strengthened hygiene measures in the case of established environmental contamination and awareness of long incubation periods where low dose contamination may be a driving factor for transmission.
